# Capitellid connections: contributions from neuromuscular development of the maldanid polychaete *Axiothella rubrocincta *(Annelida)

**DOI:** 10.1186/1471-2148-10-168

**Published:** 2010-06-08

**Authors:** Nora Brinkmann, Andreas Wanninger

**Affiliations:** 1 Department of Biology, Research Group for Comparative Zoology, University of Copenhagen, Universitetsparken 15, DK-2100 Copenhagen, Denmark

## Abstract

**Background:**

Numerous phylogenetic analyses on polychaete annelids suggest a taxon Capitellida that comprises the three families Maldanidae, Arenicolidae and Capitellidae. Recent molecular studies support the position of the Echiura, traditionally ranked as a separate phylum, within the capitellids. In order to test the robustness of this molecular-based hypothesis we take a different approach using comparative analyses of nervous and muscle system development in the maldanid *Axiothella rubrocincta*. Employing immunocytochemistry in combination with confocal laserscanning microscopy, we broaden the database on capitellid organogenesis, thereby incorporating classical histological data in our analysis. Besides assessing possible shared features with the echiurans, we also discuss the variability of neural and muscular characters within the Capitellida.

**Results:**

The scaffold of the adult central nervous system, which is already established in early developmental stages of *Axiothella*, consists of cerebral commissures that give rise to simple circumesophageal connectives with fused ventral and dorsal roots and a single ventral neurite bundle. From the latter arise segmental neurites that innervate the peripheral bodywall. Since there is no observable regular pattern, and individual neurites are lost during ontogeny, their exact arrangement remains elusive. The pharynx is encircled by a prominent stomatogastric nerve ring, with a pair of anterior and lateral proboscis neurites directly connecting it to the central nervous system. One pair of ventral and one pair of dorsal longitudinal muscles form the earliest rudiments of the bodywall musculature in late larval stages, while a continuous layer of circular muscles is lacking throughout ontogeny.

**Conclusions:**

Comparative neurodevelopmental analysis of capitellid and echiuran species reveals several common characters, including simple circumesophageal connectives, a single fused ventral nerve strand, and a stomatogastric ring nerve, that support a close relationship of both taxa, thus corroborating recent molecular phylogenetic analyses. The data on myogenesis show that four longitudinal muscle bands most likely represent an ancestral character not only for the Capitellida, but for the Annelida in general. Whether or not circular muscles are part of the annelid groundpattern remains uncertain.

## Background

The Maldanidae, also referred to as 'bamboo worms', comprise a group of deposit-feeding polychaete annelids that live in tubes composed of bottom material. They are usually considered related to the Arenicolidae and Capitellidae, and these three families are grouped together in the taxon Capitellida [1]. Recent molecular analyses have confirmed the established hypothesis of a close relationship between the Maldanidae and the Arenicolidae (lugworms) [2-4] and have repeatedly found indications that Echiura, a hotly debated group that has been traditionally ranked as a separate phylum, nests within the capitellid polychaetes [2-7]. This novel view on the phylogenetic position of the echiurans is further supported by morphological studies on neurogenesis [8-10]. In this context, investigation of the maldanid species *Axiothella rubrocincta *not only offers an opportunity to assess the ingroup variability of neural characters within the Capitellida but also allows to compare neurogenesis and nervous system organization with those data that recently have become available for echiurans. This serves as an independent test of the molecular data which propose the placement of the echiurans within the Capitellida.

Apart from the relevance of maldanids for the evolution of neural characters, *Axiothella *may also aid in casting light on the ancestral state of muscular systems in annelids and lophotrochozoans as a whole. The musculature of the Capitellida comprises a closed outer layer of circular fibers similar to that of clitellate oligochaetes [11]. However, in contrast to the latter group, recent studies have shown that circular muscles are only weakly developed or even absent in most polychaete taxa [11,12], and it has been argued that absence of circular muscles represents the plesiomorphic state for the entire Annelida [11]. Therefore, the presence of closed circular muscles in the Maldanidae represents a striking exception that deserves further investigation.

The systematics of the monophyletic Maldanidae is primarily based on external morphological features of the head, pygidium, and setae [13,14]. The maldanid ingroup relationships, as well as the monophyly of the individual subtaxa, are still unresolved [14,15]. Most studies of the internal morphology of maldanid polychaetes have focused on members of the subfamily Euclymeninae [13], to which also the investigated species, *Axiothella rubrocincta*, belongs. In particular, the comprehensive investigations of Pilgrim [16-21] serve thereby as a basis for comparison of our data on neuro- and myogenesis. We discuss the present data in the context of a hypothesized close annelid-echiuran relationship and contribute to the discussion on ancestral bodyplan features of the Annelida. In this respect, it has to be taken into account that *A. rubrocincta *represents a sibling species complex with considerable plasticity between populations concerning reproductive mode, size, and feeding, but with no obvious morphological differences [22]. Herein, we have adopted most of Pilgrim's [19-21] designations. However, we use different terms for some neuronal structures and the anterior-most muscles in the head region due to incongruency of the macro-anatomical data described by Pilgrim, which are based on light microscopy, and our confocal microscopy data.

## Results

### General development

**Figure 1:**
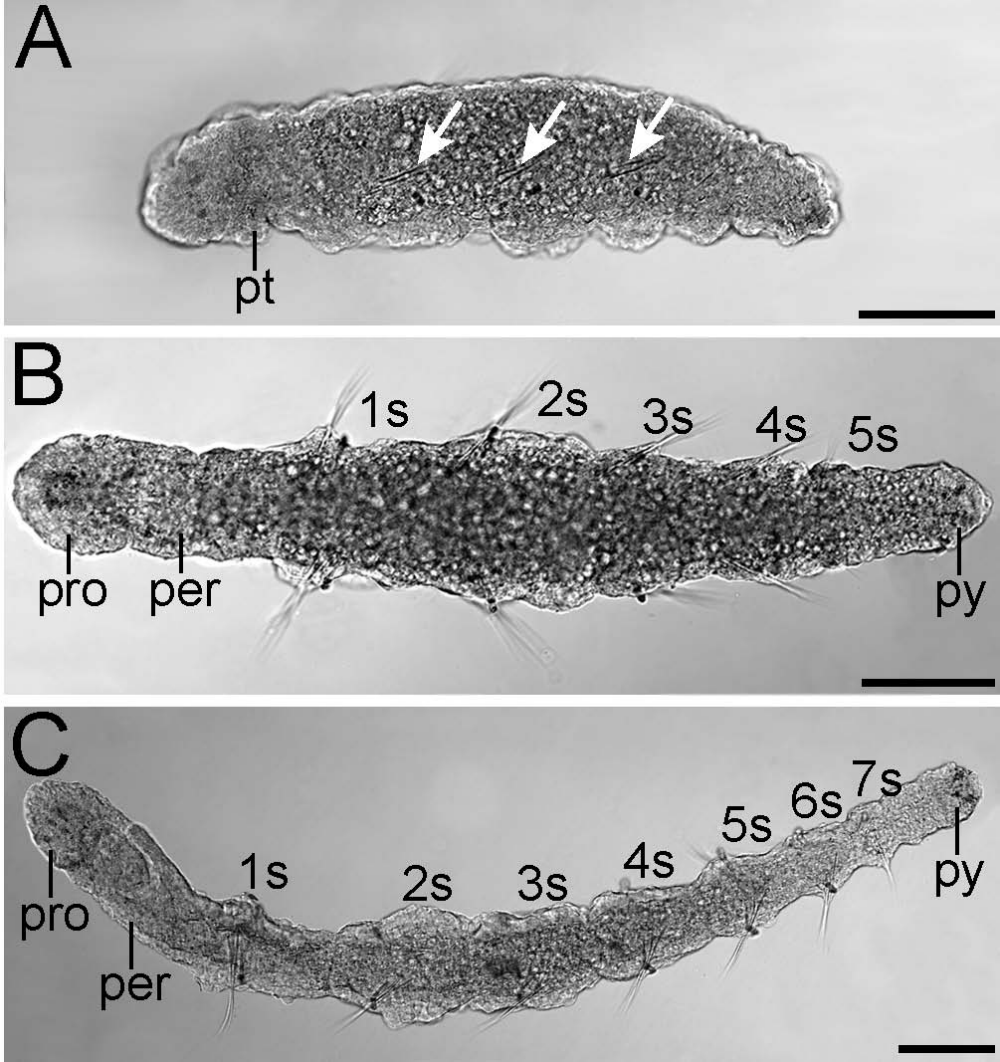
**Development of *Axiothella rubrocincta***. Light micrographs of developmental stages. Anterior is to the left. A is a lateral view, B and C are ventral views. Scale bars equal 100 μm. (A) Larva with three clearly differentiated setigers (arrows) and prototroch (pt). (B) 5-setiger juvenile (1s-5s) with differentiated prostomium (pro), peristomium (per), and pygidium (py). (C) 7-setiger juvenile.

*Axiothella rubrocincta *offspring develop inside a protective mucous cocoon, whereby development of the ciliated prototroch and telotroch shortly before the initiation of segmentation demarcates the onset of larval life. The juvenile phase starts with shedding of the proto- and telotroch at the onset of metamorphosis, i.e., at the 5-setiger stage. The cocoon contains larvae and juveniles of different stages; therefore, chronology of developmental events can only be assessed on the basis of morphological characters such as the number of setigers (setae-bearing segments), rather than by absolute time values after fertilization (Figure 1).

**Figure 2:**
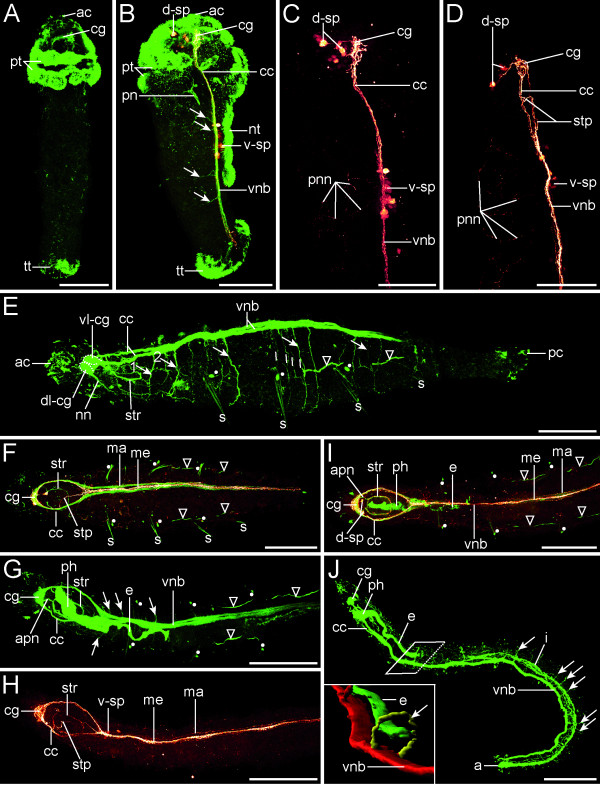
**Neurogenesis in *Axiothella rubrocincta***. Confocal micrographs showing tubulinergic (green) and serotonergic (red) immunoreactivity. Anterior faces upwards (A-D) or leftwards (E-J). Scale bars: 150 μm (A-B), 75 μm (C-E), 150 μm (F-J). (A-C) Late larva. (A) Dorsal view with cerebral ganglion (cg) established. (B) Right lateral view. Circumesophageal connectives (cc) link the ventral neurite bundle (vnb), with associated ventral perikarya (v-sp) and segmental neurites (arrows), to the cerebral ganglion (cg) and the dorsal perikarya (d-sp). (C) Enlarged anterior part of B with peripheral neural network (pnn). (D-E) 4-setiger stage, right-lateral view. (D) Enlarged anterior part of E. A stomatogastric projection (stp) branches off from the right connective (cc). (E) The cerebral ganglion (cg) shows a ventral (vl-cg) and a dorsal lobe (dl-cg). Two segmental neurites (1 and 2, arrows) innervate the head. Up to four neurites (lines) are present in the following segments. A stomatogastric nerve ring (str) surrounds the pharynx. (F) 4-setiger stage, ventral view. The ventral neurite bundle (vnb) comprises a median (me) and a paired main (ma) strand. (G-J) 7-setiger juvenile, ventral view in G-I and right-lateral view in J. (G) Note the anterior proboscis neurites (apn). (H) Same individual as in G. (J) The segmental neurites (arrows) form a ring [inset; 3D reconstruction; *red *(vnb): ventral neurite bundle, *green *(e): esophagus, *yellow *(arrow): ring neurite]. anus (a), apical cilia (ac), intestine (i), nephridioducts (triangles), neurotroch (nt), nuchal neurite (nn), pharynx (ph), posterior cilia (pc), protonephridium (pn), prototroch (pt), telotroch (tt), setae (s), uncini (dots).

The anterior region of early larvae is covered by short cilia. When the larva starts to elongate posteriorly, three ciliated bands start to differentiate: a broad prototroch which blends into the cilia of the apical plate, a neurotroch, and a telotroch (Figure 2A, and 2B). Slight invaginations of the epidermis demarcate the borders of the first three segments, and setal bundles develop pairwise in the middle of each segment (Figure 1). Moreover, the peristomium (= asetigerous first segment) and prostomium (= non-segmental, anterior-most region) differentiate anteriorly, together with the pygidium (non-segmental part) in the posterior body. Posterior to the prototroch and lateral to the ventral midline, a ciliated structure, that most likely represents a protonephridium, is visible (Figure 2B). Subsequently, a fourth setigerous segment appears posterior to the third, and approximately at the same time a pair of ventral uncini forms in each of these setigers (Figure 2E-F). Most of the ciliated regions, including the prototroch, have already been reduced at this stage, except for a few apical and posterior cilia (Figure 2E). Meanwhile, two pairs of nephridia have formed in the 4-setiger individuals. At first, the ciliated nephridioducts extend over the second/third, and over the third/fourth setiger, respectively (Figure 2E). Later on, a third and fourth pair of nephridia develop posteriorly, the body elongates further, and the digestive tract starts to form. In the 7-setiger juveniles the pharynx surrounds the mouth and extends along the entire length of the peristomial segment, followed by the esophagus in the first setiger (= second segment) (Figure 2G, and 2J). As pharynx we term the anterior-most part of the non-muscular foregut, in accordance with the descriptions of developmental stages of *Axiothella mucosa *by Bookhout and Horn [23] (Note: the term "pharynx" has been variously defined in the past [see, e.g., [24-27]]). The intestine stretches from the third setiger to the anus in the pygidium (Figure 2J). The prostomium and peristomium fuse and together form the head.

### Neurogenesis as revealed by anti-tubulin immunoreactivity

**Figure 3:**
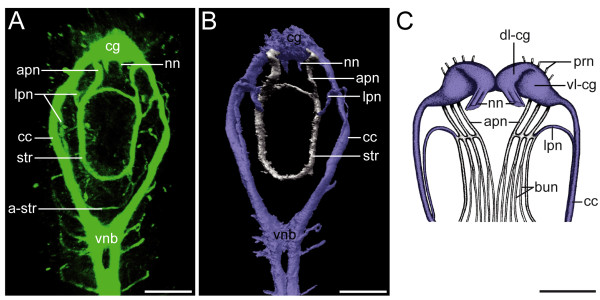
**Central and stomatogastric nervous system of the head region of *Axiothella rubrocincta***. Anterior faces upwards. Ventral view of a 7-setiger juvenile in A-B and dorsal view of an adult in C. The signal of the pharyngeal cilia has been omitted for clarity in A-B. Corresponding neuronal structures in B and C are colored blue. Scale bars equal 40 μm (A-B) and 280 μm (C), respectively. (A) Confocal micrograph showing tubulin immunoreactivity. The stomatogastric nerve ring (str) is connected via a pair of anterior proboscis neurites (apn) to the cerebral ganglion (cg) and via a pair of lateral proboscis neurites (lpn) to the circumesophageal connectives (cc) that pass into the ventral neurite bundle (vnb). In addition, an accessory stomatogastric nerve ring (a-str) is present posteriorly. (B) 3D reconstruction based on the CLSM image stack of the individual shown in A. (C) Semi-schematic representation based on histological sections, modified after Pilgrim [21]. Note the adapted designation of neuronal structures, the lack of a stomatogastric nerve ring (str), the presence of three instead of one anterior proboscis neurite (apn), the additional prostomial nerves (prn), the nuchal nerve (nn), and the nerves to the buccal epithelium (bun). The cerebral ganglion is differentiated into a dorsal (dl-cg) and a ventral lobe (vl-cg).

Apart from the external and internal cilia, antibodies against α-acetylated tubulin label microtubules of neuronal processes in developmental stages of *Axiothella rubrocincta*. Compared to the serotonin and FMRFamide stainings, this antibody allows the most detailed description of neuronal structures in the investigated species. Primarily, and shortly after the demarcation of the first three segments, the developing cerebral ganglion is labeled in late larval stages (Figure 2A-B). The cerebral commissures are densely packed and give rise to simple circumesophageal connectives whose ventral and dorsal roots are almost completely fused. Both circumesophageal strands together form a neuronal loop that extends from the cerebral ganglion into the anterior hyposphere, where the two connectives converge and pass into the ventral neurite bundle (Figure 2B). We use herein this term instead of "ventral nerve cord", because immunocytochemical analyses do not unequivocally allow discrimination between individual axons and dendrites, as is possible by transmission electron or light microscopy. The ventral neurite bundle in 3-setiger larvae consists of two main strands that lie close together and extend along the midventral line to the telotroch (Figure 2B). Later on, a third, median strand is visible in the tubulin staining, but labeling is obscured by a strong serotonergic signal (Figure 2F). Several small neurites emanate laterally from the ventral neurite bundle and innervate the bodywall of the three newly formed setigerous segments (Figure 2B). In the 4-setiger stage, the neuropil of the cerebral ganglion differentiates into a dorsal and a ventral lobe on both sides of the body. From each dorsal lobe arises a nuchal neurite that extends posteriorly to the dorsal epidermis (Figure 2E). In addition, the stomatogastric nervous system is established in developmental stages with four setigers, whereby a prominent stomatogastric neurite encircles the pharynx. This oral ring neurite is at first open on its anterior side but closes with the formation of additional segments. One pair of anterior proboscis neurites connects the stomatogastric neurite to the cerebral ganglion (Figure 2G and 2I). Furthermore, a lateral proboscis neurite branches off on each side of the stomatogastric ring nerve. These two lateral proboscis neurites are directed anteriorly before they turn back, run posteriorly, and fuse with the circumesophageal connectives (Figure 3A and 3B).

Apart from that, the number and arrangement of segmental neurites, which branch off the ventral neurite bundle, changes considerably between the 4- and 7-setiger stage. At first, two major segmental neurites are visible in the head region (Figure 2E). In most of the following setigers, approximately four segmental neurites are present in the posterior part of the segments (Figure 2E). Most of these segmental neurites appear to be ring neurites (Figure 2J, inset). Their exact arborization patterns are elusive, since their arrangement is different in the various segments. Moreover, the segmental neurites are partly reduced during development. In particular, in the anterior segments only a few small and irregularly distributed neurite branches are visible in the 7-setiger stage (Figure 2G and 2J). In the posterior four segments, however, various segmental neurites are still present. Some of these are located at the segmental borders, whereas others appear to be positioned at intersegmental furrows in the epidermis (Figure 2J).

### Serotonergic nervous system

**Figure 4:**
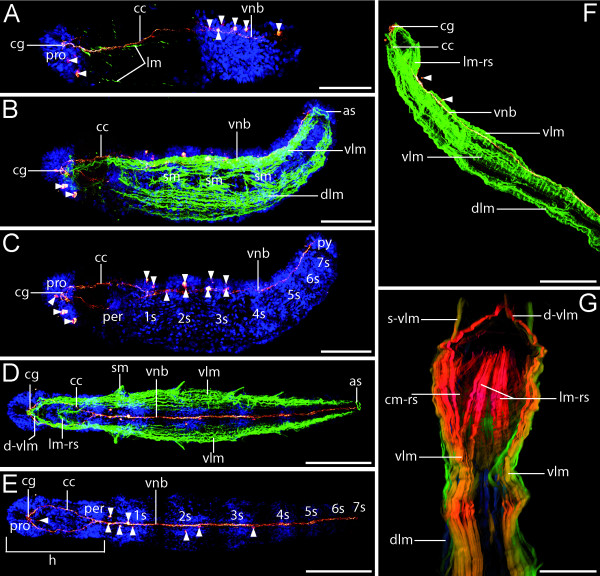
**Neuromuscular development in *Axiothella rubrocincta***. Confocal micrographs showing serotonergic immunoreactivity (red), muscles (green), and cell nuclei (blue). Anterior is to the left (A-E) or up (F-G). Scale bars: 75 μm (A-C), 135 μm (D-E), 150 μm (F), 55 μm (G). (A) Pre-segmental larva, left lateral view, right side is omitted. The cerebral ganglion (cg) with associated dorsal serotonergic perikarya (arrowheads), the circumesophageal connectives (cc), and the ventral neurite bundle (vnb) with linked ventral serotonergic perikarya (arrowheads) have already formed. The first longitudinal muscle fibers (lm) are located in a dorso- and ventro-lateral position. (B) 4-setiger stage, right lateral view. A dorsal (dlm) and ventral (vlm) longitudinal muscle band, three groups of setal muscles (sm), and an anal sphincter (as) are present. (C) Same individual as in B. (D) 5-setiger juvenile, ventral view. The anterior diagonal (d-vlm) and the longitudinal retractor sheath muscles (lm-rs) of the pharynx are derived from the ventral longitudinal muscles (vlm). (E) Same individual as in D. The body is elongated and the setigers (1s-7s) differentiate behind the head (h) region from anterior to posterior. (F-G) 7-setiger juvenile. (F) Right lateral view. Note the pair of ventral (vlm) and the single dorsal (dlm) longitudinal muscle strands. (G) Ventral view, depth-coded confocal image. The circular (cm-rs) and longitudinal (lm-rs) muscles of the retractor sheath form a basket-like structure. Anteriorly, straight ventral longitudinal muscles (s-vlm) are present in addition to the diagonal ventral longitudinal muscles (d-vlm). peristomium (per), prostomium (pro), setigers (1s-7s), pygidium (pyg).

Throughout the entire development of *A. rubrocincta*, the neurotransmitter serotonin is generally present in all major structures of the central nervous system, such as the cerebral ganglion, the circumesophageal connectives, and the almost fused ventral neurite bundle (Figure 2 and 4). Prior to the formation of the first three setigerous segments in larval stages, the circumesophageal connectives extend over more than one third of the whole body length. Serotonergic perikarya are associated with both the cerebral ganglion and the ventral neurite bundle (Figure 4A). In 3-setiger stages, three to five unipolar serotonergic perikarya are located dorsal to the cerebral commissures (Figure 2B and 2C). However, in later developmental stages only two of these cells are visible (Figure 2D and 2I; Figure 4C). In addition, up to nine perikarya form a cluster of cells in the anterior-most part of the ventral neurite bundle (Figure 2B). The staining of these serotonin-positive cell bodies is not consistent, though, and the number of labeled cells varies among individuals of the same developmental stage. In specimens with three or four setigers, a network of neurites innervates the periphery of the lateral bodywall (Figure 2C and 2D). Some of these neurites are co-localized with tubulinergic segmental neurites. There are no serotonergic neurites associated with the ciliated bands. Instead, approximately two serotonergic perikarya are connected to the ventral neurite bundle in each of the anterior segments. The ventral neurite bundle consists of one median longitudinal neurite with a high serotonin content and two small lateral neurites that correspond to the main neurites in the tubulin staining (Figure 2F, H-I). In some individuals a peculiar neuronal projection of the right circumesophageal connective is directed towards the stomodeal region (Figure 2D, F, H). Interestingly, this process is not labeled with antibodies against α-acetylated tubulin (Figure 2F). In the 7-setiger stage, the stomatogastric nerve ring and partly its connective fibers to the central nervous system exhibit serotonin immunoreactivity (Figure 2H and 2I). Along the single ventral neurite bundle, perikarya are only labeled in the peristomium and in the first setiger (Figure 2H; Figure 4F).

### FMRFamidergic nervous system

**Figure 5:**
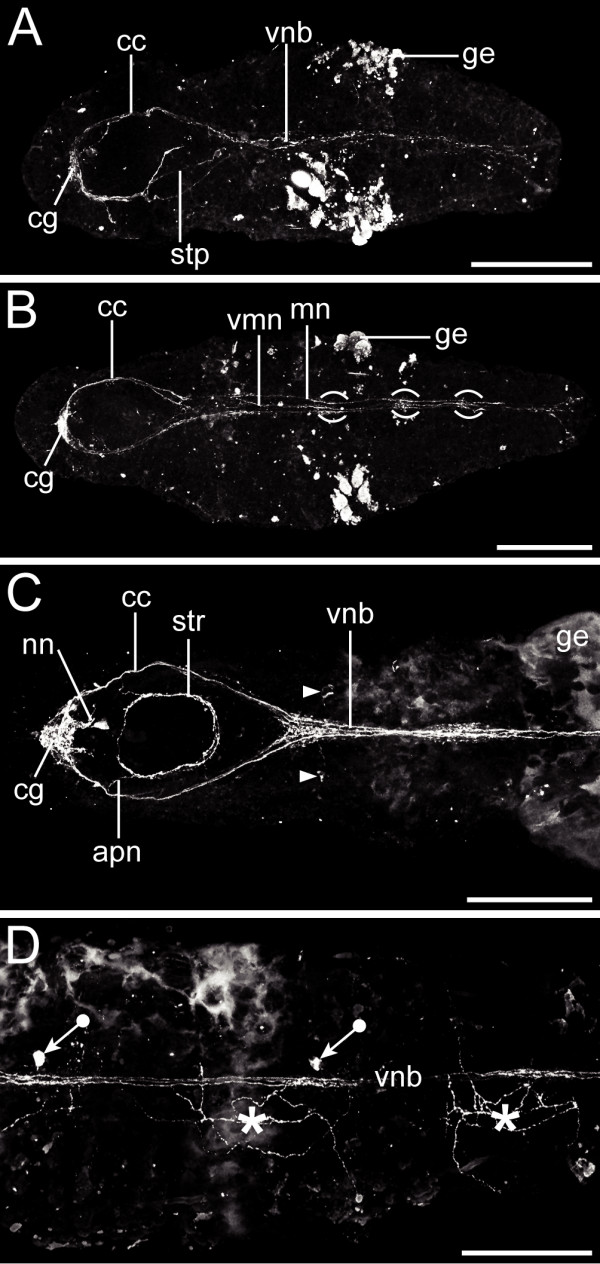
**FMRFamidergic neurogenesis in *Axiothella rubrocincta***. Anterior is to the left, ventral view. Scale bars equal 125 μm (A), 140 μm (B), and 70 μm (C-D), respectively. (A) Pre-segmental larval stage. The circumesophageal connectives (cc), the cerebral ganglion (cg), the stomatogastric connection (stp), and the ventral neurite bundle (vnb) are already established. The glandular epidermis (ge) exhibits intensive background staining. (B) 3-setiger larva. The ventral neurite bundle has differentiated into a median (me) and two main neurites (ma). The FMRFamidergic signal is particularly prominent in the mid-segmental region (brackets). (C-D) 7-setiger juvenile. (C) Immunoreactivity in the head region. Note the stomatogastric nerve ring (str), the paired anterior proboscis neurites (apn), and the dorsal cerebral processes, which most likely represent nuchal neurites (nn). Two FMRFamidergic perikarya (arrowhead) are connected to the anterior-most part of the ventral neurite bundle. (D) Immunoreactivity in the mid-body region. FMRFamidergic perikarya (tagged arrows) are connected to the ventral neurite bundle (vnb) in the third and fourth setiger. On the dorsal side of the fourth and fifth setiger a peripheral neurite plexus is present (asterisk).

In general, labeling of FMRFamide greatly resembles the results obtained for serotonin. However, due to the fact that FMRFamide is also present in the glandular epidermis, the stainings show an intensive background signal in the lateral regions of the trunk (Figure 5). At first, in late larval stages with three setigers, FMRFamide is present in the cerebral ganglion, the circumesophageal connectives, and the ventral neurite bundle. In addition, the stomatogastric projection of the circumesophageal connective is labeled (Figure 5A). Shortly afterwards, three neuronal strands, one median and two lateral, are differentiated in the ventral neurite bundle. Thereby, the FMRFamide signal is particularly prominent at the level of the setae-bearing notopodia (Figure 5B). In juveniles with seven segments, the neuropil of the cerebral ganglion shows two dorsal neuronal processes that most likely correspond to the tubulinergic nuchal neurites. In addition, the stomatogastric nerve ring and two lateral proboscis neurites are present. Moreover, FMRFamide-positive cells are connected to the anterior-most part of the ventral neurite bundle on either side (Figure 5C). In the posterior segments an FMRFamidergic peripheral plexus innervates the dorsal side of the body and single, large perikarya are arranged metamerically along the ventral neurite bundle (Figure 5D).

### Myogenesis

In pre-segmental larval stages, the first F-actin staining labels few, very delicate muscle fibers of the bodywall. The most prominent ones are oriented in a longitudinal direction and have a ventro- or a dorso-lateral position (Figure 4A). In larvae with three setigers, these longitudinal muscles form very broad muscle bands. Thus, one pair of ventral and one pair of dorsal longitudinal muscles extend from the peristomium to the posterior-most part of the body, where an anal sphincter is visible in the pygidium (Figure 4B and 4D). The labeled prostomial and peristomial muscles arise from anterior elongations of the longitudinal muscles. Laterally, setal muscles are attached to the base of the setal sheath (Figure 4B). In 5-setiger individuals, the median layer of both ventral longitudinal muscles tapers towards the prostomial tip, forming the anterior diagonal muscles, whereas the innermost layer is directed towards the mouth (Figure 4D). The latter composes the longitudinal retractor sheath muscle of the pharynx. Later on, the bucco-pharyngeal musculature exhibits a basket-like structure comprising in addition circular retractor sheath muscles. The dorsal portion of the ventral longitudinal muscles extends straight towards the anterior pole of the prostomium (Figure 4G). Interestingly, a continuous sheath of circular bodywall muscles is lacking throughout development of *Axiothella*, and the four longitudinal muscle bands do not form a closed muscle layer.

## Discussion

### Development and structure of the nervous system in Capitellida

Immunocytochemical data on polychaete neurogenesis remain scarce and are mostly restricted to isolated developmental stages. At present, there are only few studies that document the neuronal differentiation for complete developmental series and they focus on polychaetes with an indirect mode of development [28-31]. The two classical TEM-based studies on species of the Capitellida likewise offer only limited insights. The first is restricted to the 3-setiger larva of *Arenicola cristata *(Arenicolidae), whereas the second one describes different developmental stages up to metamorphosis in *Capitella capitata *(Capitellidae) [32,33]. In both studies the presented ultrastructural data are only superficially interpreted with respect to gross morphology.

One of the most prominent features of trochozoan larvae is the ciliated prototroch and its underlying serotonergic nerve ring. The latter was most likely already present in the last common ancestor of the lophotrochozoans [34,35]. Although developmental stages of *Axiothella rubrocincta *possess a ciliated prototroch, a corresponding serotonergic innervation was not found in the present study, thus corroborating earlier studies on the benthic larvae of *Arenicola *[32]. Accordingly, it appears likely that the larvae of *Axiothella *may have secondarily lost not only the serotonergic innervation of the prototroch, but maybe the entire prototroch nerve as such.

Elements of the adult central nervous system, such as the cerebral ganglion, the circumesophageal connectives, and the ventral neurite bundle, are established at a very early stage of *Axiothella *development. This simple and essentially adult organization of the central nervous system in larval stages corresponds to the neuronal arrangement described for the benthic larva of *Arenicola *and other direct developing polychaete annelids [32,36]. One characteristic feature during the observed developmental period, however, is the apparently unpaired ventral neurite bundle that does not show a primarily dichotomous organization in *Axiothella*. By contrast, the larvae of *Arenicola *possess a broad ventral neurite bundle [32]. In the genus *Capitella*, two different conditions have been documented, namely a penta-neural organization in *C. capitata *and two separate axonal tracts in *C. teleta *[36-38]. This variety in the neuronal composition of the ventral neurite bundle in developmental stages is also known from other polychaete larvae and may either indicate distant phylogenetic relationships or merely reflect the recently suggested general wide plasticity of polychaete neural patterning and nervous system anatomy [31]. Apart from that, the general arrangement of the central nervous system is consistent with previous investigations of the adult maldanid and arenicolid neuroanatomy [21,39-42]. For example, no indications of ganglionic segmentation have been found in *Arenicola *[41]. Moreover, the description of the adult ventral nerve cord in the maldanid species *Clymenella torquata *[21] can be directly correlated with the observed threefold pattern of the ventral neurite bundle with one median and two lateral main strands in the tubulin, serotonin, and FMRF-amide stainings. It is highly probable that the lateral tubulinergic strands can be assigned to a dorsally located fibrous neuropil which is separated into two parts by giant fibers that run along the ventral midline. Such multicellular giant nerve fibers have been described for several species of the Capitellida [21,39,41,43-45]. The serotonergic and FMRFamidergic median strand of the ventral neurite bundle in *Axiothella *represents most likely the precursor of such a giant nerve.

Despite these similarities in the central nervous system, conflicting views exist with regard to the stomatogastric and peripheral nervous system. The prominent stomatogastric nerve ring around the pharynx in *Axiothella rubrocincta *has not been mentioned in previous studies on the nervous system of the Maldanidae [21]. However, the neuronal fibers that connect the ring nerve to the central nervous system have been depicted and described in a similar way for the euclymenin species *Clymenella torquata *[[21]; Figure 3 present work]. The position and course of the lateral proboscis nerve in *Clymenella*, termed anterior ring nerve by Pilgrim [21], is almost identical to the one in *Axiothella*. The same holds true for the anterior proboscis nerves, which only differ in number, with one nerve being present in *Axiothella *and in several species of the genus *Clymene*, and three anterior proboscis nerves in *Clymenella *[21,46-48]. It has to be taken into account that this comparison involves on the one hand different taxa and on the other juvenile versus adult features. Given, in addition, the above stated differences in the applied methodology, it is not possible to unequivocally decide whether or not these differences indeed reflect natural conditions. However, in *Capitella*, a pair of nerves, emanating from the cerebral neuropil, encircles the mouth region, and the even more closely related taxon *Arenicola *has an additional nerve ring that surrounds the foregut at the transition between the pharynx and the esophagus [33,49]. Moreover, the descriptions of the nerves that supply the bucco-pharyngeal region in *Arenicola *agree in basic features with the documented arrangement in *Axiothella *and *Clymenella *[41,49]. Accordingly, irrespective of the varying position of the stomatogastric nerve ring, this feature is most probably part of the groundpattern of Capitellida.

The peripheral nervous system of *Axiothella *consists mainly of the segmental neurites that emerge from the ventral neurite bundle. Additional longitudinal nerve fibers have not been detected in the setigers. The arborization patterns and the exact number of the segmental neurites per setiger remain elusive. However, the arrangement of the segmental neurites does not show an obvious metameric pattern and the number of these neurites is apparently reduced during development of *Axiothella*. Similar observations have been documented previously for the adult nervous system of other maldanid taxa [21,39]. This has led to the conclusion that the nervous system of the Maldanidae shows only few signs of metamerism, namely by the presence of larger clusters of neurons opposite the parapodia and of larger nerves at the segment boundaries [21]. In *Arenicola*, however, the organization of the nervous system is very regular. A pair of nerves originates from the ventral nerve cord at the level of the borders between annuli, while opposite each setigerous annulus there are two to four pairs of nerves [41]. Slight differences in the life history traits and ecology of *Axiothella *and *Arenicola *might be the reason for the disparity in the organization of the peripheral nervous system.

### Comparative aspects of the capitellid and the echiuran nervous system

While adult echiurans show no signs of external segmentation, their larval and juvenile stages possess a clear metameric organization. Apart from the transient annulation of the body and the regular arrangement of mucous glands, this segmental organization is reflected in the structures of the nervous system during development. For example, repetitive units of serotonergic perikarya are distributed along the ventral nerve cord and precisely two pairs of peripheral nerves are associated with each ganglionic unit [8-10]. In contrast to that, there are only few signs of a metameric pattern in the maldanid *Axiothella*. According to Gamble and Ashworth [41], there is likewise no evidence for a segmental arrangement in *Arenicola*, except for the presence of giant cells in regular intervals. For the echiurans, however, it has been noted that ganglion-like groupings of perikarya are difficult to identify, since the perikarya are almost evenly distributed along the ventral nerve cord [8]. In addition, it appears that neuronal structures with a segmental arrangement such as the peripheral neurites are subsequently reduced during the ontogeny of *Axiothella*. Moreover, in *Arenicola*, these segmental neurites show indeed a regular organization with two to four pairs of nerves arising from the ventral nerve cord opposite each setigerous annulus [41]. Another indication for a possibly common structure of the nervous system of the Capitellida and the Echiura is given by serotonergic immunoreactivity in the echiuran *Bonellia viridis*, apparently revealing a stomatogastric nerve ring ([9]: Figure 4A, page 108). In addition to that, the general organization of the central nervous system in maldanid and echiuran species is largely similar with, e.g., simple circumesophageal connectives and a single ventral nerve cord in the adult. The latter results from a fusion process of an originally multi-stranded or very broad neurite bundle in the echiurans, similar to the condition found in *Arenicola *and in other genera of the Capitellidae [32,38,50]. Taken together, these similarities (Table 1) are in accordance with molecular analyses that suggest a close relationship of echiuran and capitellid taxa.

**Table 1:** Comparison of the capitellid and echiuran nervous system

	Echiura	Capitellida		
		
		*Capitella *(Capitellidae)	*Arenicola *(Arenicolidae)	*Axiothella *(Maldanidae)
repetitive units of nerve cells	+	+	+	+

metameric, peripheral neurites	+	+	+	(+)

single ventral neurite bundle	+	-*	+	+

simple circumesophageal connectives	+	?	?	+

stomatogastric nerve ring	+	?	+	+

+ present, (+) partly present, - absent, ? unknown character state

* The ventral connectives in *Capitella *are fused only in the region of the segmental ganglia. In other genera of the Capitellidae, however, there is a clear tendency towards a fused ventral nerve cord.

### Myogenesis of the bodywall musculature in Capitellida

The number and position of longitudinal muscle bands in adult polychaete annelids varies considerably among taxa [11]. However, there are only four to six longitudinal muscle bands present in most polychaetes [51]. In contrast to that, the altered arrangement in larvae of *Capitella *with eight primary longitudinal muscles most likely constitutes an exception due to secondary multiplication [52].

In the case of adult individuals of *Axiothella rubrocinta*, the number of longitudinal strands is not known. However, the closely related maldanid species *Clymenella torquata *has been depicted by illustrations of cross sections with up to six longitudinal bands that form an almost closed muscle layer [19]. In larval stages of *Axiothella*, only four delicate longitudinal muscles form the precursors of the later paired ventral and dorsal longitudinal muscle bands. Similarly, one pair of ventrolateral and another pair of dorsolateral longitudinal muscles are present in the 3-setiger larva of *Arenicola cristata *[32]. Four longitudinal muscles have also been documented in all recently investigated polychaete larvae and in developmental stages of some oligochaetes [30,53-55]. In sipunculan larvae, the first longitudinal muscle fibers likewise form a quartet and give rise to the retractor muscles of the adult [56,57]. These data strongly suggest that two pairs of primary longitudinal muscles organized in separate strands represent the plesiomorphic condition for the Capitellida and the Annelida altogether, although data on muscle development of a number of annelid taxa including the echiurans are still lacking.

Circular bodywall muscles are either poorly developed or not present in most polychaete taxa studied so far [12]. This absence of circular fibers has been interpreted as a plesiomorphic polychaete character [11]. Accordingly, the circular fibers of the capitellid species that form a closed muscle layer, similar to that of the clitellates, could represent an apomorphic feature of this group. In the investigated individuals of *Axiothella*, however, circular fibers have neither been documented in the pre-segmental larvae nor in the 7-setiger juveniles. In fact, in adult specimens of *Axiothella *the longitudinal bodywall muscles are usually more prominently developed than the circular fibers [58]. Accordingly, the lack of circular fibers during development implies that these muscles are not a larval character in *Axiothella *but that development of circular muscles is restricted to adult stages. By contrast, complete circular fibers have been interpreted as a juvenile polychaete character due to their presence in progenetic species such as *Dinophilus gryociliatus *and *Parapodrilus psammophilus *[12].

The gradual anterior-posterior development of circular muscles starts only after the initial differentiation of the longitudinal fibers in larvae of *Capitella *[52]. In the 3-setiger larva of *Arenicola*, circular fibers are present in more or less regular intervals along the longitudinal body axis [32]. Unfortunately, the dynamics involved in the formation of this circular musculature in *Arenicola *have not been studied. However, based on the gap in timing between the differentiation of longitudinal and circular muscles in *Capitella*, it has been suggested that the lack of circular fibers in polychaetes could be interpreted as a convergent reduction due to 'switching off' of the respective ontogenetic program [59]. Hence, the last common annelid ancestor might have possessed weak circular fibers which only differentiate relatively late during ontogeny. The complete layer of circular fibers, as expressed in the clitellates and capitellids, would then have evolved only in a second step to enable peristaltic movement and burrowing in firm substrate [59].

The presence of circular muscles is a shared feature of the capitellid taxa, despite the heterogeneous development of this muscle group. However, the question whether circular muscles are part of the annelid groundplan is still under discussion, also because the phylogenetic tree of the Annelida remains unresolved [2-4]. Moreover, the different myogenetic pathway of circular muscle formation in sipunculans, described recently as a synchronous-fission-type, strikingly shows the divergent ontogenetic routes that lead to the establishment of the circular layer of the bodywall musculature in annelids and their closest allies [35].

## Conclusions

Our immunocytochemical data on morphogenesis in the maldanid *Axiothella *complement previous studies and facilitate a comparison of the nervous system and musculature in capitellid polychaetes. Based on this comparative analysis, it appears that the adult nervous system of the Capitellida is secondarily reduced, comprising simple circumesophageal connectives, a characteristic stomatogastric nerve ring, and a single ventral connective as shared characters. The arrangement and number of segmental nerves and ganglion-like clusters of perikarya differ in the investigated species, possibly due to differences in the benthic life style. The data on myogenesis support the view that four longitudinal muscle bands are ancestral for Capitellida and the entire Annelida, while the presence of circular muscles is certainly a shared but not necessarily a plesiomorphic feature of the former.

The general organization of the nervous system is largely similar in capitellid and echiuran species, corroborating molecular analyses that argue for a close relationship of both taxa. However, further investigations, in particular of the neuronal connections between the stomatogastric and the central nervous system in echiuran species, are needed to substantiate this notion, since some of these common morphological traits might have been caused by convergent reduction events.

## Methods

### Animal collection and fixation

Tubes housing adult *Axiothella rubrocincta *(Johnson, 1901) were collected in the intertidal of False Bay, San Juan Island, Washington, USA, during summer 2008. The tubes contained mucous cocoons from which larvae were dissected and transferred to Petri dishes filled with Millipore-filtered seawater (MFSW). Within the cocoons, the most advanced developmental stages were found to be 7-setiger juveniles, of which some exhibit precursors of additional segments. Prior to fixation, the specimens were anesthetized with a 1:1 dilution of MFSW and MgCl_2 _(7%). They were then fixed at room temperature in 4% paraformaldehyde in 0.1 M phosphate buffer (PB) for 1.5 h, washed three times in PB, and stored at 4°C in PB containing 0.1% sodium azide (NaN_3_).

### Immunolabeling and confocal laserscanning microscopy (CLSM)

The following steps were all performed at 4°C. Antibody staining was preceded by tissue permeabilization for 1 h in 0.1 M PB with 0.1% NaN_3 _and 0.1% Triton X-100 (PTA), followed by overnight incubation in block-PTA [6% normal goat serum (Sigma-Aldrich, St. Louis, MO, USA) in PTA]. The primary antibodies, polyclonal rabbit anti-serotonin (Zymed, San Francisco, CA, USA, dilution 1:800), polyclonal rabbit anti-FMRFamide (Chemicon, Temecula, CA, USA, dilution 1:400), and monoclonal mouse anti-acetylated α-tubulin (Sigma-Aldrich, dilution 1:1000), all in block-PTA, were either applied separately or in a mixed cocktail for 24 h. Subsequently, the specimens were rinsed in block-PTA with three changes over 6 h and incubated in a mixture of 4'6-diamidino-2-phenyl-indole [DAPI (Invitrogen, Eugene, OR, USA)], secondary fluorochrome-conjugated antibodies [goat anti-rabbit FITC (Sigma-Aldrich), dilution 1:400; goat anti-rabbit Alexa Fluor 594 (Invitrogen), dilution 1:1000; goat anti-mouse FITC (Sigma-Aldrich), dilution 1:400] and, for F-actin visualization, Alexa Fluor 488 phalloidin (Molecular Probes, Eugene, OR, USA; dilution 1:40) in block-PTA overnight. Finally, the specimens were washed three times in PB without NaN_3 _and were directly mounted in Fluoromount G (Southern Biotech, Birmingham, AL) on glass slides. A minimum of 10 immunolabeled specimens per developmental stage was analyzed for each antibody. Approximately 65 image stacks of optical sections were recorded as Z-wide-projections with 0.1-0.5 μm step size using a Leica DM IRE2 fluorescence microscope equipped with a Leica TCS SP 2 confocal laserscanning unit (Leica, Wetzlar, Germany). Setae are visible in the tubulin scans due to autofluorescence. Images were processed with Adobe Photoshop CS3 to adjust contrast and brightness and were arranged into figure plates using Adobe Illustrator CS3 (Adobe Systems, San Jose, CA, USA). The three-dimensional computer reconstructions were generated with the imaging software Imaris v. 5.5.3 (Bitplane, Zürich, Switzerland) using surface rendering algorhithms.

## Abbreviations

1s-7s: setigers; a: anus; ac: apical cilia; apn: anterior proboscis neurite; as: anal sphincter; a-str: accessory stomatogastric nerve ring; bun: nerves of the buccal epithelium; cc: circumesophageal connective; cg: cerebral ganglion; cm-rs: circular muscle of the retractor sheath; dl-cg: dorsal lobe of the cerebral ganglion; dlm: dorsal longitudinal muscle bundle; d-sp: dorsal serotonergic perikarya; d-vlm: diagonal ventral longitudinal muscle; e: esophagus; ge: glandular epidermis; h: head; i: intestine; lm: longitudinal muscle fiber; lm-rs: longitudinal muscle of the retractor sheath; lpn: lateral proboscis neurite; ma: main strand; me: median strand; nn: nuchal neurite; nt: neurotroch; pc: posterior cilia; per: peristomium; ph: pharynx; pn: protonephridium; pnn: peripheral network of neurites; prn: prostomial nerves; pro: prostomium; pt: prototroch; py: pygidium; s: setae; sm: setal muscles; stp: neuronal stomatogastric projection; str: stomatogastric nerve ring; s-vlm: straight ventral longitudinal muscle; tt: telotroch; vl-cg: ventral lobe of the cerebral ganglion; vlm: ventral longitudinal muscle bundle; vnb: ventral neurite bundle; v-sp: ventral serotonergic perikarya.

## Authors' contributions

NB performed research, analyzed data and drafted the manuscript. AW designed and coordinated research and contributed to writing of the manuscript. Both authors conceived the study, read, and approved the final version of the manuscript.
